# Knowledge, Attitude, and Awareness of Adolescents on the Emergency Management of Traumatic Dental Injuries

**DOI:** 10.3390/dj14030182

**Published:** 2026-03-19

**Authors:** Neetha Shenoy, Supreet Kaur, Sandya Kini K, Neeta Shetty, Vani Lakshmi R

**Affiliations:** 1 Department of Conservative Dentistry and Endodontics, Manipal College of Dental Sciences, Manipal Academy of Higher Education, Manipal 576104, Karnataka, India; supreet.jagmohan@manipal.edu (S.K.); sandya.kini@manipal.edu (S.K.K.); 2 Private Practitioner, Mangaluru 575001, Karnataka, India; neeta.shetty@manipal.edu; 3 Department of Health Technology and Informatics, Prasanna School of Public Health, Manipal Academy of Higher Education, Manipal 576104, Karnataka, India; vani.lakshmi@manipal.edu

**Keywords:** adolescents, avulsion, emergencies, awareness, attitude, sports injuries

## Abstract

**Background/Objectives**: Traumatic dental injuries (TDIs), particularly avulsion, require immediate and appropriate first aid to ensure favorable outcomes. Adolescents are often the first responders during school or sports activities, yet their preparedness remains unclear. This study aimed to assess the knowledge, attitude, and awareness of adolescents regarding the emergency management of TDIs. **Methods**: A descriptive cross-sectional survey was conducted among 400 adolescents aged 15 to 18 years from four randomly selected colleges in Mangaluru, Karnataka. A structured, validated 16-item questionnaire assessed demographic characteristics and domains of knowledge (6 items), attitude (6 items), and awareness (6 items). Data was analyzed using descriptive statistics, chi-square tests, and one-way ANOVA. **Results**: The mean knowledge score was 2.50 ± 1.04 (95% CI: 2.40–2.60), indicating limited knowledge of dental trauma management; only 26.3% of participants recognized that avulsed permanent teeth can be replanted and **7%** identified an appropriate storage medium. The mean attitude score was comparatively high (4.38 ± 1.12; 95% CI: 4.27–4.49), with 88.8% of students willing to assist an injured peer, reflecting a large attitude–knowledge discrepancy (Cohen’s d = 1.47). The mean awareness score was 2.24 ± 1.24 (95% CI: 2.12–2.36), indicating limited awareness of preventive practices, including low mouthguard use (11.5%). Shapiro–Wilk testing confirmed non-normal distribution of KAA scores (*p* < 0.05); accordingly, non-parametric analyses showed no significant differences across schools, academic streams, gender, or education level (Kruskal–Wallis and Mann–Whitney U tests; *p* > 0.05). **Conclusions**: Despite favorable attitudes toward assisting peers, adolescents demonstrated limited knowledge and awareness regarding the emergency management and prevention of traumatic dental injuries, particularly in tooth replantation, appropriate storage media, and mouthguard use, highlighting the need for targeted, school-based dental first-aid education programs.

## 1. Introduction

Traumatic dental injuries (TDIs) represent a significant global oral health challenge, particularly among children and adolescents who are highly vulnerable to accidents during school and recreational activities [1]. Recent epidemiological reports estimate that between 15% and 20% of school-aged children experience trauma to permanent dentition, most commonly affecting the anterior teeth [2,3]. Consistent with global estimates, Indian studies report a substantial burden of traumatic dental injuries, with prevalence ranging from 12% to 30% among school-going children, and higher rates observed in urban and semi-urban regions with greater participation in outdoor activities [4].

TDIs such as avulsion and complicated crown fractures can result in pain, esthetic impairment, functional difficulty, and considerable psychological distress if not managed promptly [5]. Among these, the prognosis of avulsed permanent teeth is especially time-sensitive and depends greatly on immediate and appropriate first-aid measures [6]. The recent International Association of Dental Traumatology (IADT 2020) guidelines emphasize that the first few minutes after avulsion are critical and that early intervention by a bystander can markedly improve outcomes [7].

Despite the need for prompt emergency management, preparedness for dental trauma remains inadequate, highlighting critical gaps in training and awareness. While previous studies have primarily focused on teachers, parents, and healthcare professionals, data on adolescents aged 15–18 years—who are often the first responders during peer-related injuries—remain limited [8,9,10,11]. This age group is particularly vulnerable due to increased physical activity, participation in sports and recreational activities, interpersonal conflicts, and a higher risk of road traffic accidents. In India, this gap is particularly evident, as most research targets younger children or adult caregivers, leaving the preparedness of middle adolescents largely unexplored [2,4,12].

Preparedness for managing traumatic dental injuries (TDIs) in adolescents is effectively assessed using the knowledge–attitude–awareness (KAA) framework, as knowledge underpins appropriate first-aid actions, while attitudes and awareness influence willingness, confidence, and timely response [13,14,15]. In Karnataka, particularly in coastal districts such as Mangaluru where adolescents frequently engage in contact sports and outdoor recreational activities, data on preparedness for managing TDIs are lacking [15,16]. Identifying gaps in knowledge, attitudes, and awareness in this context is essential for developing targeted, school-based training and preventive strategies. Therefore, this cross-sectional study aimed to assess the knowledge, attitude, and awareness of adolescents aged 15–18 years in Mangaluru, Karnataka, regarding the emergency management of traumatic dental injuries.

## 2. Materials and Methods

### 2.1. Ethical Approval

The study protocol was reviewed and approved by the Institutional Ethics Committee (Reference No. 18062). Administrative permission was obtained from each participating school. Written informed assent was obtained from all students along with parental consent. Participation was voluntary, and strict anonymity and confidentiality were ensured throughout data handling and reporting.

### 2.2. Study Design and Setting

A descriptive cross-sectional questionnaire survey was conducted among adolescents aged 15–18 years from four pre-university colleges (equivalent to Classes XI and XII) in Mangaluru district, Karnataka. It was conducted and reported in accordance with the STROBE (Strengthening the Reporting of Observational Studies in Epidemiology) guidelines (Supplementary S1).

### 2.3. Sample Size Determination

The sample size was calculated using a single proportion formula with a 95% confidence level and an absolute precision of 5%. In the absence of prior studies reporting the prevalence of knowledge on dental trauma emergency management in the target population, an expected proportion (*p*) of 0.50 was assumed as a conservative estimate to maximize the required sample size.

$$n=\frac{Z^{2}p(1-p)}{d^{2}}$$

with Z = 1.96, *p* = 0.50, and d = 0.05, the minimum required sample was 384. To compensate for non-response (approximately 4% based on pilot study), the target sample was set at 400 students.

### 2.4. Participants and Sampling Procedure

Cluster random sampling was employed, with each pre-university college considered as one cluster. The official district list of all pre-university colleges served as the sampling frame. In the first stage, four colleges were selected using a computer-generated randomization sequence. In the second stage, all eligible students aged 15–18 years from the selected colleges were approached and surveyed during on-site data collection. Data was collected during September 2024 and November 2024 in classroom settings under investigator supervision. Students who provided informed assent and parental consent and were able to read English or Kannada were included. Exclusion criteria included prior dental, medical, or first-aid training, absence on the day of data collection, and questionnaires with more than 20% missing responses (if any).

### 2.5. Questionnaire Development

A structured questionnaire consisting of 16 items (Supplementary S2) was developed by adapting elements from previously published questionnaires used in studies on emergency management of dental trauma [11,12,13,14]. Minor linguistic and contextual modifications were made to align with regional terminology and student comprehension levels.

The questionnaire consisted of two sections. The first section collected demographic information using four items assessing age, gender, class, and academic stream. The second section comprised 16 closed-ended questions assessing knowledge, attitude, and awareness regarding the emergency management of traumatic dental injuries. This section included six knowledge questions, six awareness-related questions, and four attitude questions, one of which contained three sub-items. For scoring purposes, the three sub-items were considered as independent attitude items, resulting in a total of six attitude items.

### 2.6. Validation and Pilot Testing

Content validity was evaluated by a panel of three experts in pediatric dentistry and public health using the Content Validity Index (CVI). Items with a CVI < 0.80 were revised or removed. A pilot study was conducted among 30 students from a nonparticipating pre-university college to evaluate the clarity, comprehension, and operational feasibility of the questionnaire. The pilot was administered using the same standardized procedures as the main study, including identical instructions, classroom setting, administration time, and supervision by the investigator. Students completed the questionnaire individually, and their feedback on wording, flow, and response options was recorded. Based on the pilot, minor modifications were made for clarity and language refinement. Data from the pilot study was excluded from the final analysis. The finalized questionnaire demonstrated acceptable internal consistency (Cronbach’s alpha = 0.72).

### 2.7. Data Collection Procedure

The survey was administered in classroom settings during designated periods. Standardized verbal instructions were provided by the investigator before distribution. Students completed the questionnaire individually in approximately 15–20 min. The investigator remained present to ensure standardized administration and to prevent peer influence. Completed questionnaires were collected immediately. No personal identifiers were recorded on the questionnaires. Each response was assigned a unique numerical code prior to data entry to ensure anonymity. The coded data were stored in password-protected electronic files accessible only to the investigators and were used exclusively for research purposes.

### 2.8. Bias Control

To minimize potential sources of bias, several study-specific measures were implemented. All eligible students present in the selected classes were surveyed to reduce selection bias. Questionnaires were anonymous and assigned unique numerical codes to prevent identification of participants. Teachers were not present during questionnaire administration, and completed questionnaires were collected immediately to minimize peer discussion and social desirability influences. Standardized verbal instructions were delivered by the investigator across all study sites, and questionnaires were administered within a fixed time frame in classroom settings to reduce procedural variability. The questionnaire assessed students’ current knowledge and intended responses to dental trauma scenarios rather than recollection of past events, thereby reducing potential recall bias.

### 2.9. Scoring of Knowledge, Attitude, and Awareness

Knowledge was assessed using six objective items related to the emergency management of dental trauma. Each correct response was scored as 1 and each incorrect response as 0, yielding a total knowledge score ranging from 0 to 6, with higher scores indicating better knowledge. Awareness was assessed using six dichotomous (Yes/No) items related to exposure, experience, and preparedness for dental trauma. Affirmative responses were scored as 1 and negative responses as 0, resulting in a cumulative awareness score ranging from 0 to 6. Attitude was assessed using six items reflecting students’ perceptions, intentions, and willingness to respond to dental trauma scenarios. Positive attitude responses were scored as 1 and negative responses as 0, yielding a total attitude score ranging from 0 to 6.

### 2.10. Statistical Analysis

Data were entered into Microsoft Excel and analyzed using SPSS version 21.0 (IBM Corp., Armonk, NY, USA). Descriptive statistics, including frequency, percentage, mean ± standard deviation, and median (interquartile range), were used to summarize demographic characteristics and questionnaire responses.

Normality of the score distributions was assessed using the Shapiro–Wilk test; the data were found to be non-normally distributed. Accordingly, non-parametric tests were applied for inferential analysis. Comparisons between two independent groups (gender and education level) were performed using the Mann–Whitney U test, while comparisons among more than two groups (stream) were carried out using the Kruskal–Wallis test. Associations between categorical variables were evaluated using the Chi-square test. Correlations between age, awareness, attitude, and knowledge scores were assessed using Spearman’s rank correlation coefficient. All statistical tests were two-tailed, and a *p*-value < 0.05 was considered statistically significant.

## 3. Results

### 3.1. Participant Characteristics

A total of 400 students participated in the study. The age distribution ranged from 15 to 18 years (16.7 ± 0.63 years), with the majority being 17 years old (57.8%). There were 211 males (52.8%) and 189 females (47.3%). The demographic details are presented in Table 1. The overall response rate was 100%, with 400 complete questionnaires included in the final analysis.

### 3.2. Awareness Related to Dental Trauma

**Figure 1:**
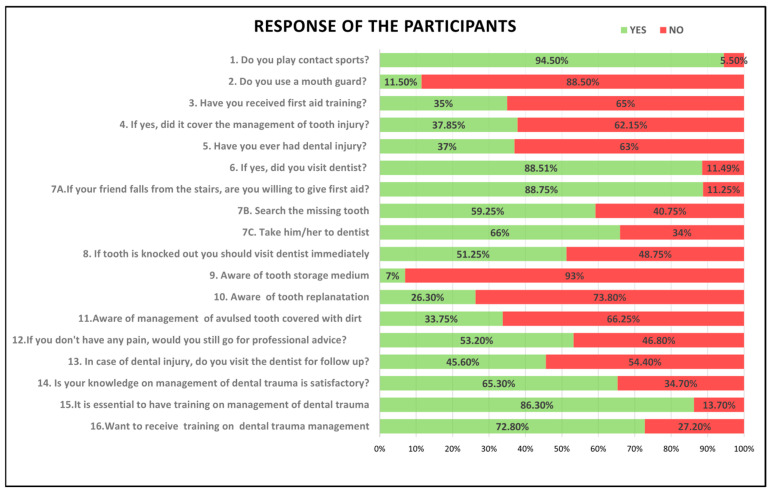
Graphical representation of the participants’ responses to questions on knowledge, awareness, and attitude.

Figure 1 shows the response of the participants. In total 378 students (94.5%) reported involvement in contact sports; however, only 46 students (11.5%) used a mouthguard during sports activities. A total of 140 students (35%) had received general first-aid training, of whom 53 students (37.85%) reported that the training included topics on dental trauma.

A history of dental injury was reported by 148 students (37%), and among them, 131 students (88.51%) sought dental consultation following the injury. Table 2 summarizes the responses related to knowledge, awareness and attitude.

### 3.3. Attitude Toward Emergency Management

Most participants (*N* = 355; 88.8%) stated they would assess injuries and provide first aid to a classmate who had fallen during school hours. Additionally, 237 students (59.25%) reported that they would search for a missing tooth if avulsion was suspected, and 264 students (66.0%) indicated that they would take the injured classmate to a dentist. In total, 291 students (72.8%) expressed willingness to attend an educational program on dental trauma management (Table 2).

### 3.4. Knowledge Regarding Emergency Management of Dental Trauma

Only 105 students (26.3%) were aware that an avulsed permanent tooth can be re-planted. Awareness regarding the correct management of a soiled avulsed tooth was reported by 135 students (34.5%). Knowledge of the appropriate storage medium for transporting an avulsed tooth was low, with only 28 students (7%) identifying a suitable medium. The mean knowledge score of the participants was 2.19 ± 1.28.

### 3.5. Comparison of Knowledge Scores Between Schools

The mean knowledge score of the participants was 2.50 ± 1.04 out of a maximum possible score of 6. The mean attitude score was 4.38 ± 1.12, while the mean awareness score was 2.24 ± 1.24. These findings indicate higher attitude scores than knowledge and awareness scores among the participants (Table 3).

Normality testing using the Shapiro–Wilk test revealed that the score distributions were non-normal (*p* < 0.05); therefore, non-parametric tests were employed for further analysis. Knowledge, attitude, and awareness scores were compared across gender and education level using the Mann–Whitney U test, while comparisons among streams/schools were conducted using the Kruskal–Wallis test. No statistically significant differences were observed between schools for knowledge, attitude, or awareness scores (*p* > 0.05) (Table 4).

Spearman’s rank correlation analysis demonstrated weak correlations among the study variables. A weak positive correlation was observed between attitude and knowledge scores (ρ = 0.180, *p* < 0.001), and between age and attitude score (ρ = 0.099, *p* < 0.05). No meaningful correlations were found between awareness score and either knowledge or attitude scores (*p* > 0.05), indicating that awareness, attitude, and knowledge largely functioned as independent constructs in this population.

## 4. Discussion

This study evaluated the knowledge, attitude, and awareness of adolescents regarding the emergency management of traumatic dental injuries (TDIs). The findings highlight substantial knowledge gaps despite generally positive attitudes and moderate awareness among participants.

In the present study, the majority of students (94.5%) reported participation in contact sports, confirming substantial exposure to TDI risk. Despite this, only 11.5% reported using mouthguards, reflecting poor preventive practices. This discrepancy suggests that high exposure to injury risk is not matched by adoption of protective behaviors among adolescents in this setting. Similar low compliance has been reported globally and is commonly attributed to limited awareness [17] and lack of enforcement [18]. The European Association for Sports Dentistry advocates education and mandatory custom-made mouthguards (CSM) use in high-risk sports to reduce TDI prevalence [19], indicating that policy-level reinforcement, in addition to awareness, may be required to improve compliance.

In our study, only 35% of the students reported receiving general first-aid training, and among these, just 37.85% had received instructions related to dental trauma management. Similar deficits were documented by Pithon et al. [20] and Alyahya et al. [21] underscoring a persistent omission of dental trauma content in first-aid education. This finding suggests that even when adolescents are exposed to first-aid training, dental emergencies are often deprioritized in favor of life-threatening conditions such as bleeding, fractures, or cardiopulmonary resuscitation (CPR) [22,23]. The absence of dental trauma modules within existing school health programs may therefore contribute directly to the low knowledge scores (2.50 ± 1.04) observed in this study.

A considerable proportion (37%) of participants had experienced dental injury themselves, reflecting the high local burden of TDIs. However, 88.5% of those who sustained trauma sought dental consultation, suggesting satisfactory awareness of the need for professional management once injury occurs. These figures are encouraging when compared to other studies, where fewer adolescents reported seeking immediate dental care [3,20]. Nevertheless, in our study, prior experience with dental injury did not correspond to higher knowledge scores, implying that experiential exposure alone does not translate into better preparedness.

Adolescents exhibit varied attitudes towards traumatic dental injuries (TDIs), influenced by psychosocial factors, lifestyle, and the perceived impact on their quality of life [11,24]. In the present study, attitude scores were comparatively high (mean 4.38 ± 1.12), indicating a strong willingness to help and seek care. Most respondents (88.8%) stated that they would provide first aid to a classmate who sustained an injury at school, demonstrating a commendable helping attitude and readiness to act. However, only 59.25% would actively search for a missing tooth if avulsion was suspected, indicating basic awareness of urgency but limited understanding of correct handling or replantation procedures. This attitude–knowledge gap is clinically significant, as willingness to help is not necessarily accompanied by correct action. According to recent IADT guidelines, immediate replantation of an avulsed permanent tooth at the site of injury offers the best prognosis [18,23]; failure to recognize or act upon this step can lead to irreversible tooth loss and long-term functional and psychosocial consequences [23,24,25].

A higher proportion (66%) of participants reported that they would take the injured person to a dentist, aligning with findings from Wang et al. [5] and Young et al. [11], reported strong referral intent but limited procedural knowledge. Encouragingly, nearly three-fourths (72.8%) expressed willingness to attend an educational program on dental trauma management, reinforcing their receptivity to structured learning interventions. This receptiveness highlights a critical opportunity for intervention, as positive attitudes, also reported by Alyahya et al. [21] among parents and adolescents with access to educational materials, may facilitate effective knowledge transfer when supported by structured educational initiatives.

**Figure 2:**
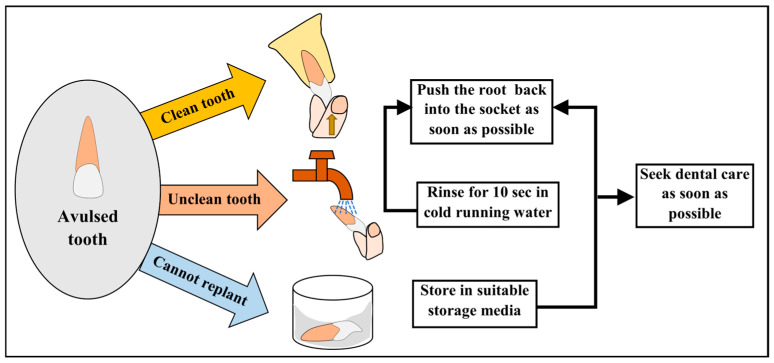
Schematic representation of emergency management of an avulsed permanent tooth.

Awareness that an avulsed permanent tooth can be replanted was reported by only 26.3% of students, consistent with previous Indian studies [2,14], and reflected in the low mean awareness score of 2.24 ± 1.24. Additionally, misconceptions regarding cleaning a soiled avulsed tooth were evident, with many students endorsing harmful practices such as scrubbing or rinsing under tap water. These findings demonstrate a critical disconnect between general awareness and accurate procedural knowledge, reinforcing the need for targeted, skill-based education aligned with IADT guidelines [18,23]. Figure 2 outlines the schematic representation of handling an avulsed tooth during replantation.

Knowledge of appropriate storage media was particularly deficient, with only 7% of participants identifying a suitable option. This represents one of the most critical gaps, as extra-alveolar storage conditions strongly influence periodontal ligament cell viability and replantation success. Similar deficiencies have been reported across diverse populations [3,11,13,21,26,27], suggesting a widespread lack of dissemination of simple, actionable dental first-aid information. The low awareness of readily available media such as milk or saline, despite their accessibility in school environments, indicates that existing health education frameworks inadequately emphasize practical emergency steps [14].

Overall, the findings indicate that adolescents possess positive attitudes and moderate awareness but insufficient procedural knowledge regarding TDI management. Given that most dental injuries in this age group occur in school or sports settings, interventions should be strategically targeted to these environments. Structured, school-based educational programs focusing on avulsion recognition, immediate re-plantation, and appropriate storage media have been shown to significantly improve preparedness [28]. Incorporating dental first-aid training into school curricula, reinforcing mouthguard use through policy and supervision, and using visual aids and peer-led demonstrations may directly address the specific knowledge gaps identified in this study. Emphasizing practical, contextually feasible measures—such as the use of milk or saline as storage media—can further enhance real-world applicability and response effectiveness [28,29,30,31].

### 4.1. Strengths and Limitations

This study is among the few from India to focus exclusively on middle adolescents (15–18 years), a group frequently underrepresented in traumatic dental injury research. The inclusion of multiple colleges and an adequately powered sample enhances the robustness of the findings. However, the cross-sectional design and reliance on self-reported responses may introduce reporting and social desirability biases and preclude causal inference. As the study was conducted in a single coastal district of Karnataka, regional variations in school resources, exposure to health education, and sports culture may act as cultural or educational confounders, thereby limiting generalizability. Additionally, although the questionnaire was validated, measurement constraints inherent to structured surveys may have influenced the depth of assessed knowledge and awareness. Although cluster sampling was used with pre-university colleges as the sampling units, the statistical analyses were conducted at the individual level without explicit adjustment for clustering. As a result, the variance estimates may be underestimated, and the reported *p*-values should be interpreted with caution. Future studies should consider using multilevel or cluster-robust analytical approaches to account for intra-cluster correlation.

### 4.2. Future Directions

Future research should focus on evaluating the effectiveness of school-based dental first-aid modules, assessing long-term knowledge retention, and studying the impact of hands-on training on behavioral outcomes. Incorporating digital learning tools and gamified educational interventions may further improve engagement and learning among adolescents. Policies promoting the mandatory use of mouthguards during contact sports should also be explored as preventive measures to reduce TDI incidence.

## 5. Conclusions

In the present study, adolescents aged 15–18 years showed critical gaps in knowledge of traumatic dental injury management, especially regarding replantation, handling, and storage of avulsed teeth, with a low mean knowledge score of 2.50 ± 1.04. In contrast, attitudes toward assisting injured peers were positive, with a mean attitude score of 4.38 ± 1.12, and awareness levels were modest (mean awareness score 2.24 ± 1.24), revealing a clear attitude–knowledge–awareness gap. These findings indicate that adolescents are motivated but insufficiently equipped to respond effectively to dental trauma. Incorporating targeted, skill-oriented dental first-aid training into school health programs, along with reinforcement of preventive strategies such as mouthguard use in contact sports, may substantially improve preparedness and outcomes following dental injuries.

## Figures and Tables

**Table 1:** Demographic characteristics of the participants.

Variables		N (%)
Q1. Age (Years)	15	5 (1.3%)
	16	128 (32%)
	17	231 (57.8%)
	18	36 (9%)
Q2. Gender	Male	211 (52.8%)
	Female	189 (47.3%)
Q3. Education	Class XI	60 (15%)
	Class XII	340(85%)
Q4. Stream	Arts	26 (6.5%)
	Science	72 (18%)
	Commerce	302 (75%)

**Table 2:** Responses of the participants to the questionnaire.

**Sl No.**	**Awareness Questions**	**Yes**	**No**
Q1.	Do you play contact sports?	378 (94.5%)	22(5.5%)
Q2.	Do you use a mouth guard?	46 (11.5%)	354(88.5%)
Q3.	Have you received first aid training?	140 (35%)	260(65%)
Q4.	If yes, did it cover the management of tooth injury? (N = 140)	53 (37.85%)	87(62.15%)
Q5.	Have you ever had dental injury?	148 (37%)	252(63%)
Q6.	If yes did you visit dentist? (N = 148)	131(88.51%)	17(11.49%)
**Sl No.**	**Attitude Questions**	**Yes**	**No**
Q7A.	During school hours, your classmate falls from the stairs. Would you like to look for any injury in the body and give first aid?	355 (88.75%)	45 (11.25%)
Q7B.	After controlling bleeding, if you notice that the front tooth is missing, would you search for the tooth immediately?	237(59.25%)	163 (40.75%)
Q7C.	Should you take him/her to dentist?	264 (66%)	136 (34%)
Q8.	Do you think that your knowledge of emergency management is satisfactory?	261 (65.25%)	139 (34.75%)
Q9	Do you think it is essential to have an educational program regarding the management of dental trauma?	345(86.25%)	55 (13.75%)
Q10	Would you like to receive short training on how to manage dental trauma cases?	291 (72.8%)	109 (27.2%)
**Sl No.**	**Knowledge Questions**	**Correct (%)**	**Incorrect (%)**
Q11.	In case of dental injury, if you don’t have any pain, would you still go for professional advice?	212 (53.0)%	188(47.0%)
Q12.	Visit to the dentist for follow up	264 (66%)	136 (34%)
Q13.	Ideal time for the treatment (MCQ: answer “Immediately”)	205 (51.25%)	195 (48.75%)
Q14.	An avulsed permanent tooth can be replanted	105 (26.25%)	295 (73.75%)
Q15.	Management of avulsed tooth covered with dirt.	135 (34.75%)	265 (66.25%)
Q16.	Storage medium	28 (7%)	372 (93%)

**Table 3:** Descriptive statistics of knowledge, attitude, and awareness scores by gender, education, and stream.

**Knowledge Scores**			
**Levels**	**Mean ± SD**	**Median (Q1,Q3)**	***p* Value**
Male	2.46 ± 1.05	2.00 (2.00,3.00)	<0.001
Female	2.53 ± 1.07	3.00 (2.00,3.00)	<0.001
Class XI	2.40 ± 0.79	2.00 (2.00,3.00)	<0.001
Class XII	2.51 ± 1.08	3.00 (2.00,3.00)	<0.001
Science	2.38 ± 0.98	2.00 (2.00,3.00)	<0.001
Commerce	2.53 ± 1.08	3.00 (2.00,3.00)	<0.001
Arts	2.42 ± 1.21	2.00 (2.00,3.00)	<0.001
**Attitude Scores**			
**Levels**	**Mean ± SD**	**Median (Q1,Q3)**	***p* Value**
Male	4.36 ± 1.12	4.00 (4.00,5.00)	<0.001
Female	4.41 ± 1.13	4.00 (4.00,5.00)	<0.001
Class XI	4.48 ± 1.07	4.50 (4.00,5.00)	<0.001
Class XII	4.36 ± 1.13	4.00 (4.00,5.00)	<0.001
Science	4.17 ± 1.16	4.00 (4.00,5.00)	<0.001
Commerce	4.45 ± 1.11	4.00 (4.00,5.00)	<0.001
Arts	4.19 ± 1.13	4.00 (4.00,5.00)	<0.001
**Awareness Scores**			
**Levels**	**Mean ± SD**	**Median (Q1,Q3)**	***p* Value**
Male	2.25 ± 1.28	2.00 (1.00,3.00)	<0.001
Female	2.23 ± 1.20	2.00 (1.00,3.00)	<0.001
Class XI	2.20 ± 1.29	2.00 (1.00,3.00)	<0.001
Class XII	2.25 ± 1.23	2.00 (2.00,3.00)	<0.001
Science	2.36 ± 1.24	3.00 (2.00,3.00)	<0.001
Commerce	2.23 ± 1.24	2.00 (2.00,3.00)	<0.001
Arts	2.04 ± 1.22	1.50 (1.00,3.00)	<0.001

**Table 4:** Kruskal–Wallis and Mann–Whitney U test results for knowledge, attitude, and awareness scores.

Score	Kruskal–Wallis			Mann–Whitney U (Gender)		Mann–Whitney U (Education)	
	χ^2^	df	*p*-Value	U	*p*-Value	U	*p*-Value
Awareness Score	1.45	2	0.485	19,796	0.897	9951	0.754
Attitude Score	4.68	2	0.096	19,438	0.652	9682	0.515
Knowledge Score	1.55	2	0.460	19,279	0.549	9496	0.371

## Data Availability

The original contributions presented in this study are included in the article and Supplementary Materials. Further inquiries can be directed to the corresponding author.

## Supplementary Materials

The following supporting information can be downloaded at: https://www.mdpi.com/article/10.3390/dj14030182/s1, S1: Annexure 1: STROBE Statement—Checklist of items that should be included in reports of cross-sectional studiestle; S2: Annexure 2: Questionnaire to assess the knowledge, attitude and awareness of Pre-university students on dental trauma.

## Abbreviations

The following abbreviations are used in this manuscript:

TDIs	Traumatic dental injuries
IADT	International Association of Dental Traumatology
KAA	Knowledge–Attitude–Awareness
HBSS	Hank’s Balanced Salt Solution
CSM	Custom-made mouthguards
CPR	Cardiopulmonary resuscitation
