# Supplementary material for: Knowledge, Attitude, and Awareness of Adolescents on the Emergency Management of Traumatic Dental Injuries
Source: Dent J (Basel). 2026 Mar 19;14(3):182. doi: 10.3390/dj14030182 (PMC13025531; doi:10.3390/dj14030182)
Supplement: Supplementary file 1 [file dentistry-14-00182-s001.zip › dentistry-4026242-supplementary.pdf]

**S1. Annexure1: STROBE Statement—Checklist of items that should be included in reports of *cross-sectional studies***

| | Item No | Recommendation | Page no. |
|---|---|---|---|
| **Title and abstract** | 1 | (*a*) Indicate the study's design with a commonly used term in the title or the abstract | 1 |
| | | (*b*) Provide in the abstract an informative and balanced summary of what was done and what was found | 1 |
| **Introduction** | | | |
| Background/rationale | 2 | Explain the scientific background and rationale for the investigation being reported | 2 |
| Objectives | 3 | State specific objectives, including any prespecified hypotheses | 2 |
| **Methods** | | | |
| Study design | 4 | Present key elements of study design early in the paper | 2-4 |
| Setting | 5 | Describe the setting, locations, and relevant dates, including periods of recruitment, exposure, follow-up, and data collection | 3-4 |
| Participants | 6 | (*a*) Give the eligibility criteria, and the sources and methods of selection of participants | 3-4 |
| Variables | 7 | Clearly define all outcomes, exposures, predictors, potential confounders, and effect modifiers. Give diagnostic criteria, if applicable | 3-4 |
| Data sources/ measurement | 8* | For each variable of interest, give sources of data and details of methods of assessment (measurement). Describe comparability of assessment methods if there is more than one group | *4* |
| Bias | 9 | Describe any efforts to address potential sources of bias | 4 |
| Study size | 10 | Explain how the study size was arrived at | 3 |
| Quantitative variables | 11 | Explain how quantitative variables were handled in the analyses. If applicable, describe which groupings were chosen and why | 4 |
| Statistical methods | 12 | (*a*) Describe all statistical methods, including those used to control for confounding | 4 |
| | | (*b*) Describe any methods used to examine subgroups and interactions | 4 |
| | | (*c*) Explain how missing data were addressed | 4 |
| | | (*d*) If applicable, describe analytical methods taking account of sampling strategy | 3-4 |
| | | (*e*) Describe any sensitivity analyses | Not applicable |
| **Results** | | | |
| Participants | 13* | (a) Report numbers of individuals at each stage of study—eg numbers potentially eligible, examined for eligibility, confirmed eligible, included in the study, completing follow-up, and analysed. | 5 |
| | | (b) Give reasons for non-participation at each stage | Not applicable |
| | | (c) Consider use of a flow diagram | Not applicable |
| Descriptive data | 14* | (a) Give characteristics of study participants (eg demographic, clinical, social) and information on exposures and potential confounders | 6 (Table 1) |
| | | (b) Indicate number of participants with missing data for each variable of interest | Not applicable |

| | | | |
|---|---|---|---|
| Outcome data | 15* | Report numbers of outcome events or summary measures | 5-8 |
| Main results | 16 | (*a*) Give unadjusted estimates and, if applicable, confounder-adjusted estimates and their precision (eg, 95% confidence interval). Make clear which confounders were adjusted for and why they were included | Not applicable |
| | | (*b*) Report category boundaries when continuous variables were categorized | Not applicable |
| | | (*c*) If relevant, consider translating estimates of relative risk into absolute risk for a meaningful time period | Not applicable |
| Other analyses | 17 | Report other analyses done—eg analyses of subgroups and interactions, and sensitivity analyses | 5-8 |
| **Discussion** | | | |
| Key results | 18 | Summarise key results with reference to study objectives | 5-8 |
| Limitations | 19 | Discuss limitations of the study, taking into account sources of potential bias or imprecision. Discuss both direction and magnitude of any potential bias | 10 |
| Interpretation | 20 | Give a cautious overall interpretation of results considering objectives, limitations, multiplicity of analyses, results from similar studies, and other relevant evidence | 9-10 |
| Generalisability | 21 | Discuss the generalisability (external validity) of the study results | 9-11 |
| **Other information** | | | |
| Funding | 22 | Give the source of funding and the role of the funders for the present study and, if applicable, for the original study on which the present article is based | 11 |

*Give information separately for exposed and unexposed groups.

**Note:** An Explanation and Elaboration article discusses each checklist item and gives methodological background and published examples of transparent reporting. The STROBE checklist is best used in conjunction with this article (freely available on the Web sites of PLoS Medicine at http://www.plosmedicine.org/, Annals of Internal Medicine at http://www.annals.org/, and Epidemiology at http://www.epidem.com/). Information on the STROBE Initiative is available at www.strobe-statement.org.

## S2. Annexure 2: Questionnaire to assess the knowledge, attitude and awareness of Pre-university students on dental trauma.

**Part 1 Demographic Characteristics of the participants**

1. Age:
2. Gender: Male/Female
3. Education: Class XI/ Class XII
4. Stream: Arts/Science/Commerce

**Part 2: Questions on Knowledge, attitude and awareness of dental trauma (Select the most appropriate answer)**

1. Do you play contact sports? Yes/No
2. Do you use a mouth guard? Yes/No
3. Have you received first aid training? Yes/No
4. If yes, did it cover the management of tooth injury? Yes/No
5. Have you ever had dental injury? Yes/No
6. If yes, did you visit dentist? Yes/No
7. During school hours, your classmate falls from the stairs.

a. Would you like to look for any injury in the body and give first aid? Yes/No
b. After control of bleeding, will you search for the lost tooth? Yes/No
c. Should you take him/her to dentist? Yes/No

8. Do you think that your knowledge of emergency management is satisfactory? Yes/No
9. Do you think it is essential to have an educational program regarding the management of dental trauma? Yes/No
10. Would you like to receive short training on how to manage dental trauma cases? Yes/No

11. In case of dental injury, if you don't have any pain, would you still go for professional advice? Yes/No
12. In case of dental injury, do you visit the dentist for follow up? Yes/No
13. How urgent do you think that immediately you should seek professional help if permanent tooth is knocked out?

a. Immediately
b. within 30 minutes
c. Within 1 hour
d. Not urgent at all.

14. Would you replant (put back) the tooth in its socket? Yes/No
15. What would you do if you decide to replant a tooth back into its socket but it has fallen onto the ground and is covered with dirt?

a. Scrub the tooth gently with a toothbrush
b. Rinse the tooth under tap water.
c. Put the tooth straight back into the socket without doing other things.
d. Others (antiseptic/alcohol)
e. Do not know

16. If you have to take the tooth to the dentist, how would you transport the tooth?

a. Wet cotton
b. Ice
c. Water
d. Milk
e. Special solution/ Transport medium
f. Place it under tongue/ buccal sulcus
